# Challenges for Adult Undocumented Immigrants in Accessing Primary Care: A Qualitative Study of Health Care Workers in Los Angeles County

**DOI:** 10.1089/heq.2020.0036

**Published:** 2020-09-02

**Authors:** Matthew Yu, A. Taylor Kelley, Anna U. Morgan, Andrew Duong, Anish Mahajan, Jessica D. Gipson

**Affiliations:** ^1^ North Side Christian Health Center, Pittsburgh, Pennsylvania, USA.; ^2^ Department of Internal Medicine, Division of General Internal Medicine, University of Utah School of Medicine, Salt Lake City, Utah, USA.; ^3^ Informatics, Decision-Enhancement and Analytic Sciences (IDEAS) Center, VA Salt Lake City Health Care System, Salt Lake City, Utah, USA.; ^4^ Fielding School of Public Health, University of California, Los Angeles, Los Angeles, California, USA.; ^5^ Los Angeles County Department of Health Services, Harbor-UCLA Medical Center, Torrance, California, USA.

**Keywords:** undocumented immigrants, Los Angeles County, primary care, access to health care, Public Charge Rule, community health center

## Abstract

**Purpose:** Amid increasingly restrictive federal immigration and health care policies in the United States, access to health care for undocumented immigrants is highly dependent on the extent to which local and state policies and programs address the needs of this population. In Los Angeles County (LA County), home to the nation's largest undocumented immigrant population, supportive policies are in place, yet little is known about how undocumented immigrants navigate available services.

**Methods:** To gain insight into how federal, state, and local policies overlay and contribute to the experience of health care seeking among undocumented immigrants in LA County, we interviewed 19 key informant health care workers involved in the delivery of health care services, using a purposive snowball sampling approach.

**Results:** Three key themes emerged: (1) health care workers at all clinics sampled reported primary care appointments are readily available for undocumented immigrants; however, primary care services remain underutilized; (2) fear, misinformation, and misperceptions of coverage and immigration policies—most commonly related to the revised Public Charge Rule—may reduce program enrollment and access; and (3) frontline health care workers feel ill-equipped to address patient fears and misinformation.

**Conclusion:** Although county programs were perceived to improve access by covering health care costs and ensuring appointment availability, new restrictive immigration policies, such as the revised Public Charge Rule, and widespread misinformation present challenges that threaten the success of these programs. Future study to improve undocumented immigrant access to care should focus on addressing barriers resulting from these policies.

## Introduction

Undocumented immigrants face unique challenges in the U.S. health care system.^1–6^ They are ineligible for federal insurance programs in the Affordable Care Act (ACA), resulting in the lowest health care coverage rates of any segment of the U.S. population.^2,3,7^ Lack of coverage has led to harmful delays in care, increased acute care utilization, and poor health outcomes.^4–6,8–17^ In addition, recent changes and even proposed changes to immigration policies and practices have engendered fear within immigrant communities, resulting in further negative health and social impacts.^18–24^ These policies, in addition to other challenges (e.g., language, discrimination, and stigma), result in a formidable health care environment for undocumented immigrants.^1,25–27^

Exclusion of undocumented immigrants from federal health care coverage has prompted a wave of strategies and interventions at local and state levels. Most undocumented immigrants reside in metropolitan areas,^28^ and since the ACA, many of these areas have developed programs to provide reduced or no-cost primary and specialty care services to undocumented immigrant populations. For example, New York City Health and Hospitals developed sliding scale financial assistance through the Options program^29^; Grady Health in Atlanta assists undocumented residents through a Chronic Care Clinic^30^; and Dallas,^31^ Houston,^32^ and Chicago have leveraged county-based systems of care.^29^ Preliminary findings from studies in these communities demonstrate that in spite of efforts to extend public health services, the ramifications of federal policy may limit utilization of these services among undocumented immigrants.^29,33^

Los Angeles County (LA County) is home to >1 million undocumented immigrants—the largest of any county in the United States.^34^ LA County provides a safety-net system of care through the Department of Health Services (LAC-DHS), which serves all patients irrespective of ability to pay or immigration status.^35^ When the ACA reduced federal safety-net funding that covered costs for providing care to the uninsured because of expanding Medicaid eligibility among citizens and other documented people,^35^ LAC-DHS began to explore innovative approaches to primary care delivery for its residually uninsured population—an estimated 474,000 residents ineligible for coverage even after coverage expansions of the ACA went into effect.^36^ The result was the establishment of My Health LA in 2014,^37^ a program providing primary care free-of-charge to residually uninsured county residents <138% of the federal poverty level, through >200 partnering federally qualified health centers across the county.^38^ However, as of 2019, just 142,000, or ∼30% of residually uninsured residents, had enrolled in My Health LA.^37^

In early 2020, the White House amended language for the Department of Homeland Security Inadmissibility on Public Charge Grounds policy (Public Charge Rule), stating that lawfully present immigrants using social benefits, including Medicaid, may be denied permanent legal resident status.^39,40^ Furthermore, even being likely to need such benefits might disqualify immigrants from receiving permanent legal status. Understanding how these policy changes impact primary care engagement among undocumented immigrants is, therefore, essential in developing strategies to ensure equitable care and mitigate harms.

In this context, we sought to (1) understand LA County health care worker perceptions of access to primary care for undocumented immigrants and (2) identify potentially modifiable barriers to ideal utilization of care among undocumented immigrants in LA County by interviewing health care workers providing care or access services for this population during 2018–19. The Public Charge Rule change was proposed but not yet adopted at the time of the study.

## Methods

Nineteen semistructured in-depth interviews were conducted from August 2018 to January 2019 with LA County health care workers who facilitate or provide services for undocumented immigrants. Working with LAC-DHS administrative personnel, we first identified health care worker roles within county programs that have frequent regular interaction with undocumented immigrants, including patient registration, financial and access services, clinical care, federally qualified health center staff, and My Health LA enrollment and administrative staff. We next contacted six key informant health care workers to conduct initial in-depth interviews and identify navigation processes within their institutions. We then asked these individuals to identify others serving in similar roles. We recruited 13 additional participants using this snowball sampling approach (*n*=19). In total, nine agencies were represented, including My Health LA and eight clinical sites providing undocumented immigrant care: five LAC-DHS sites (two hospitals and three clinics) and three federally qualified health centers. Potentially identifying information on participants has been omitted to preserve anonymity. See Table 1.

**Table 1.** Role Descriptions of Health Care Worker Interview Participants in Los Angeles County

Respondent	Description of role	Number interviewed
CCALAC Administrative staff member	Acts as intermediary between CCALAC member clinics and local, state, and federal officials; educates decision makers about impacts of proposed legislation on underserved and/or member clinics (CCALAC represents 65 health centers and community clinics across LA County that comprise >300 clinic sites serving 1.6 million patients. The Policy and Advocacy division focuses on ensuring and sustaining health care funding for medically indigent, uninsured, and vulnerable populations served by member clinics.)	1
LAC community clinic administrative staff	Assists uninsured patients in FQHCs with eligibility counseling for Medicaid or MHLA and participation in sliding scale payment programs for outpatient care.	3
LAC community clinic Outreach and Health Education administrative staff member	Supervises health education and outreach department, with recent experience as a community health worker; assists in insurance and financial program enrollment, utilization, and retention for patients in an FQHC setting; personally assists undocumented patients with various program applications, including PRUCOL, Medicaid, and FQHC sliding-scale programs.	1
LAC-DHS Continuity Care Clinic provider	Provides clinical care to uninsured patients with outpatient clinic services who have had a recent emergency room or hospital visit and require care coordination until they can be bridged into a primary care medical home.	2
LAC-DHS Emergency Room Provider	Provides emergency room medical services in a LAC-DHS hospital.	1
LAC-DHS Office of Patient Access	Assists patients with health system navigation, including scheduling for new patient primary care, follow-up care, and specialty care; assists with linking patients between LAC-DHS hospitals and community clinics (some staff work in dual settings between MHLA and the Office of Patient Access).	
LAC-DHS Patient Financial Services Administrator and Staff	Assists patients with health service coverage options, particularly for those who are uninsured; assists with applications for Medi-Cal or other low-cost/no-cost programs when/if not eligible for Medi-Cal; assists patients with medical bills.	3
LAC-DHS Patient Registration staff administrator	Oversees clerical staff for patient registration in hospital and outpatient settings; participates in financial screening, insurance verification, and LA County program eligibility verification for enrollment in medical services.	2
MHLA Administrator	Oversees program operations, planning, and implementation of MHLA to achieve sustainability and growth (MHLA is a no-cost healthcare program for people who live LA County who are ineligible for other public health insurance programs).	2

FQHCs, federally qualified health centers; LAC-DHS, Los Angeles County Department of Health Services; MHLA, My Health LA.

Our instruments were informed by existing empirical literature, as well as first-hand clinical knowledge of the LA County system. Participants interviewed were asked to share perspectives on their roles, observations, and experiences relating to undocumented immigrants, including (1) program access and enrollment, (2) health care service navigation, and (3) patient care. Digitally recorded interviews were 30–90 min in length, conducted in a private office space by the lead author. One interview was conducted by phone per request. In three instances participants were accompanied by another staff member to augment the information. All aspects of the study received IRB approval.

Digital recordings were transcribed verbatim without personal identifiers using encrypted transcription software (Temi), then reviewed by two transcribers to ensure accuracy and quality. We analyzed the data using a modified grounded theory approach^41^ involving coding (open/axial/en vivo codes), memoing, and team meetings. Transcripts were first reviewed and coded by the lead author to identify main themes and subthemes, then iteratively reviewed with the senior author to identify and resolve discrepancies. After coding transcripts, a second round of analysis was conducted by revisiting the transcripts to identify excerpts to illustrate key themes. Finally, all findings were discussed and verified with team members to verify contextual accuracy.

## Results

Interview data from LA County health care workers revealed three key themes: (1) all eight sites reported readily available primary care appointments for undocumented immigrants in LA County; (2) despite health care workers' perceptions of clinics as “safe spaces,” primary care services remain underutilized due to fear, misinformation, and misperceptions of coverage and immigration policies; and (3) frontline health care workers feel ill-equipped to address patient fears and misinformation. Verbatim interview extracts are presented in Table 2.

**Table 2.** Verbatim Interview Extracts from Participants (*n*=19)

Theme/subtheme	Verbatim extracts
(A) Readily available primary care	
	(1) “If there's issues with your health insurance or health plan or access to Medi-Cal, we go ahead and still see [patients] for that visit. Before we used to say, ‘Oh, you don't have insurance, let's send you away or let's send you to go see a Medi-cal worker’ Where now we're trying to service the patient first so they have a better patient experience and ensure that they're seen and assessed, then work all that other stuff on the back end.”
	—*LAC-DHS Office of Patient Access staff member*
	(2) “On the same day [as registering], most of our patients are able to even do a physical. I have had patients come in, applied, see the doctor, and the dentist. All on the same day…”
	—*LA County Community Clinic MHLA and registration staff*
	(3) “The clinic has its own low-cost sliding scale program for patients that do not qualify for LAC or Federal programs. They are able to enroll the patients into these sliding scale programs first and allow them to be seen by a provider. Later on, if they qualify for a program, their co-pay can be refunded. Often, they can be seen the same day if the need is urgent. The front office staff can also look for availability in other satellite clinics.”
	—*LA County Community Clinic administrative staff*
	(4) “For those who are not out-of-country or out-of-county, who are truly undocumented LA county residents, they're actually easier to take care of because we can schedule them for [primary and specialty care] follow up [in the County system].”
	—*LAC-DHS Emergency Room Provider*
(B) Underutilization of primary care services	
Declining enrollment in LA County programs or Medicaid	(1) “We had a patient [who] said, ‘I want to cancel my Medi-Cal,’ and I respond, ‘Why would you cancel your Medi-Cal if you need it?’ (because he's having surgery sometime this week). He says, ‘Because of the new rule (Public Charge) that is coming up. I reply, ‘Oh, there's nothing in place here. It's only a proposal. You don't have to be afraid of it.’ He insisted on canceling it… He was a legal resident, not even undocumented and had his green card.”
	—*LAC-DHS Patient Financial Services staff member*
	(2) “We have heard that patients don't want to enroll in MHLA or they don't want to renew because we go into a lot of detail like, ‘where do you work and where you live and how many kids do you have?’ And I think some undocumented people in this climate are opting to not answer those questions and so they cancel.”
	—*MHLA Administrator*
	(3) “Every day, I feel like there's at least one patient that comes in that has an issue with, you know, public charging, how it might affect them or their kids. Patients are declining to apply for Medi-Cal and/or choosing not to renew their application.”
	—*LA County Community Clinic Outreach and Health Education administrative staff member*
Fear of attending clinic appointments	(4) “There was a legit drop in patients coming to the clinic because they didn't want to be standing on the bus stop or waiting around to get picked up [by ICE agents]. There was a significant no-show rate in patients that didn't come to their appointments.”
	—*LA County Community Clinic Outreach and Health Education administrative staff member*
	(5) “Since early 2017, we've been pushing out to clinics, policies and procedures. What to do when immigration agents show up at your clinic? Do your staff know what to do?…Really having to go through this whole other layer of sort of re-training if you will, to make sure everyone is aware of the protections that are in place. Doing another level of work around branding the health centers as a safe space for anyone regardless of immigration status, which is new for us.”
	—*Community Clinic Association of LA County administrative staff member*
	(6) “Here, I think it's a very safe place because they [patients] live within the community and they've been here for years. The people that continue coming to our clinic are basically family members or friends of friends that come to this clinic.”
	—*LA County Community Clinic and My Health LA registration staff*
Fear of sharing information with health care providers and programs	(7) “Because of that fear [of deportation], they might give you different information than their actual name. A different name, a different birthdate, we've had patients that actually told us later on that ‘I used my friend's name’ and we asked them, ‘Why?’… ‘Well, I was scared.’”
	—*LAC-DHS Patient Registration staff member*
	(8) “It's just their culture in the sense of, ‘The less you know about me, the safer I am at home because of my immigration status.’”
	—*LAC-DHS Patient Access Center representative*
Excessive complexity in navigating services	(9) “Patients rarely understand how to navigate the financial side of accessing healthcare outside of showing up in the emergency room”
	—*LAC-DHS Patient Financial Services administrator*
	(10) “Most people working in LAC-DHS, unless you're working in Patient Financial Services, have no idea of [patient health access programs/insurances]. They're confused when you start talking about undocumented [patients] and [health] insurance and access.”
	—*LAC-DHS Continuity Care Clinic provider*
	(11) “I'm not afraid to say I even have doctors who tell their patients come to LA County even though they're from Orange County, which is not good because you're setting them up for big charges and they're not going to qualify for anything.”
	—*LAC-DHS Emergency Room provider*
	(12) “There's definitely a huge amount [of misinformation about healthcare access options]. It has always been that those who are out-of-county/out-of-country cannot receive follow up. This policy was more blurred prior to the ACA, prior to the empanelment that declares where people should be receiving their care.”
	—*LAC-DHS Financial Services*
	(13) “Patients rarely know the difference between Medi-Cal and MHLA. This is a good thing and speaks to the lack of disparity present between the two programs.”
	—*LAC-USC Continuity Clinic Staff*
(C) Frontline staff ill-equipped to address their patients' challenges	
Frontline staff feel ill-equipped to address their patients' challenges	(1) “It's almost like playing [the game] telephone in the sense where the communication can spread quickly and then it gets diluted…down the road…it is difficult to combat what they heard from their neighbor or what they saw on TV.”
	—*Community Clinic Association of LA County administrative staff member*
	(2) “If we're not informed, we can't really help our patients or help them make an informed decision.”
	—*LA County Community Clinic Outreach and Health Education administrative staff member*
	(3) “You go into our community, [you will see] any number of places advertising ‘Notarios', and you will see the list of legal services that they are fraudulently providing our community and very affordable pricing.”
	—*LAC-DHS Continuity Care Clinic provider*
	(4) “It's complicated [in that] immigration advice needs to be provided by an immigration attorney. Clinic staff should not be trying to wade into a person's individual circumstances and provide them with any sort of guidance or advice that goes way beyond their capabilities.”
	—*Community Clinic Association of LA County Administrative staff member*

ACA, Affordable Care Act; CCALAC, Community Clinic Association of Los Angeles County; ICE, Immigration and Customs Enforcement; LA County, Los Angeles County.

### Readily available primary care

“…Before we used to say, ‘Oh, you don't have insurance, let's send you away or let's send you to go see a Medi-Cal [enroller],’ where now we're trying to service the patient first so they have a better patient experience, ensure that they're seen and assessed, then work all that other stuff on the back end.”—LAC-DHS Office of Patient Access staff member.

Participants from every clinic reported availability of appointments—most the same day (See Table 2: Quotes A1–2). Inability to pay/lack of coverage was not reported as a barrier to being able to be seen for an appointment—even for patients unwilling to apply or ineligible for Medi-Cal or My Health LA (A3).

Participants indicated health care providers and support staff are dedicated to providing high-quality equitable care and, as a matter of practice, do not ask about a patient's documentation status when providing services. Most participants reported that quality of care for undocumented immigrants was uncompromised despite fewer options for care.

Health care workers reported numerous efforts to reduce access barriers, such as the development of algorithms to prioritize primary care visits for high-risk patients after hospital and emergency room discharges, and leadership support to expand undocumented coverage programs (A4).

### Underutilization of primary care services

Participants reported several barriers to utilization of primary care services. According to participants, fears of deportation and/or unfavorable consequences from the [then proposed] Public Charge Rule change were frequent and common, even among well-informed undocumented people and their families. Excessive complexity in navigating programs and services designed around restrictive federal policies was also commonly reported.

Declining enrollment in LA County programs or Medicaid

“We had a patient [who] said, ‘I want to cancel my Medi-Cal,’ and I respond, ‘Why would you cancel your Medi-Cal if you need it?’ (he was having surgery that week). He says, ‘Because of the new rule (Public Charge) that is coming up. I reply, ‘Oh, there's nothing in place here. It's only a proposal. You don't have to be afraid of it.’ He insisted on canceling it.”—LAC-DHS Patient Financial Services staff member

Participants noted that influence from media and/or local legal advice have discouraged enrollment in public programs to minimize chances of deportation (B1). Some patients have disenrolled themselves or family members from Medi-Cal, My Health LA, and other services due to fear of the Public Charge Rule, even if it did not apply to them (local and state programs do not apply, and the Public Charge Rule change had only been proposed, not enacted) (B2–3).

### Fear of attending clinic appointments

“There was a legit drop in patients coming to the clinic because they didn't want to be standing on the bus stop or waiting around to get picked up [by U.S. Immigration and Customs Enforcement (ICE) agents]. There was a significant no-show rate…”—LA County Community Clinic Outreach and Health Education administrative staff member

Participants reported that undocumented immigrants attend clinic visits less frequently; therefore, efforts have expanded to increase engagement (B4). Fear of deportation was reported to be so prevalent that the largest consortium of federally qualified health centers in LA County produced immigration policy briefs and held training sessions for enrollment staff (B5).

In contrast, participants reported that among those who engage in care, federally qualified health centers were generally perceived as safe spaces. Participants reported undocumented immigrants are willing to disclose fears and challenges with health care workers they trust (B6).

#### Fear of sharing information with health care providers and programs

“Because of that fear [of deportation], they might give you different information than their actual name…and we asked them, ‘Why?’…‘Well, I was scared.’”—LAC-DHS Patient Registration staff member

Participants reported patients often expressed concern that ICE agents may contact them if they apply for Medi-Cal (B7). Although My Health LA is not a federal program, becoming eligible requires a completed—and rejected—application for Medi-Cal, and many undocumented immigrants choose more costly prepayment plans because less personal identifying information is required and/or they fear the Medi-Cal application itself could make them a public charge. As a result of these concerns, participants noted that undocumented patients may falsify personal information on the applications or may avoid programs for themselves if they thought their enrollment might put other family members at risk (B8).

#### Excessive complexity in navigating services

“Patients rarely understand how to navigate the financial side of accessing healthcare outside of showing up in the emergency room”—LAC-DHS Patient Financial Services administrator

Participants reported that both undocumented immigrants and many frontline clinical staff have poor access to information about health care options or the varied eligibility requirements of insurance and county programs, resulting in greater emergency room utilization and less primary care (B9–13). This was particularly true for hospital follow-up.

### Frontline staff ill-equipped to address their patients' challenges

“It's almost like playing [the game] telephone in the sense where the communication can spread quickly and then it gets diluted…down the road…it is difficult to combat what they heard from their neighbor or what they saw on TV.”—Community Clinic Association of Los Angeles County (CCALAC) administrative staff member

“If we're not informed, we can't really help our patients or help them make an informed decision.”—LA County Community Clinic Outreach and Health Education administrative staff member

Frontline health care staff observed unmet needs for patient education and navigation assistance for immigration law and health care eligibility, but felt ill-equipped to address these concerns (C1–4). In addition to word-of-mouth spread of misinformation within the community, participants also reported competing with unreliable community information sources, such as “notarios”—notaries or paralegals in Latino communities—who sometimes advertise services beyond their scope of licensure and provide inaccurate and incorrect guidance regarding health care decisions. (C1, 3). Clinic registration staff felt stretched beyond their capabilities when attempting to educate patients on the legal implications of Medi-Cal application and use, simultaneously trying to correct misconceptions and mitigate harms from false information. For example, participants reported that some undocumented immigrants would not utilize any health care services for fear of jeopardizing legal status eligibility or entry into the United States; they cited difficulties in communicating with patients to allay these concerns. Differences in language were reported to exacerbate these difficulties. In addition, participants reported that information about the nuances of different programs and eligibility criteria were not common knowledge among many within the health care system, creating another source of misinformation for patients.

## Discussion

Despite the development of programs providing primary care to undocumented immigrants in LA County, health care workers identified numerous challenges that prevent undocumented people from accessing available medical care. We situate our findings hereunder by focusing on the distinct policy, health system, and individual-level barriers for undocumented immigrants.^1,42^

At a policy level, the exclusion of coverage expansions for undocumented immigrants in the ACA has raised concern for further marginalization of this population.^2,43,44^ Even in a supportive environment such as LA County, where local programs have ensured covered primary care services are readily available for undocumented immigrants and health care workers report delivering care unaware of their patients' documentation status, restrictive immigration policies were reported to negatively affect health care seeking among undocumented immigrants. Although fear of deportation has been long-standing, participants identified the Public Charge Rule change—then proposed and now official—as another source of fear. Because the new policy may disqualify immigrants who use public benefits from obtaining legal permanent resident status—for themselves or even for native-born children—many may forego needed care.

We may well expect that individual and societal costs, suffering, and adverse outcomes resulting from delayed and foregone care will rise in LA County and elsewhere. Whereas the Public Charge Rule previously affected 3% of undocumented immigrants, the revised definition will impact as many as 47% of current undocumented immigrants.^18,45^ Important to note is that novel coronavirus disease 2019 (COVID-19) emerged just after enactment of the rule change. Although the pandemic's substantial health and financial impacts are being felt globally and nationally, there is heightened concern that current federal policies may further deter undocumented immigrants from seeking care during a critical public health emergency.^46^ Services related to the diagnosis and treatment of COVID-19 do not make an individual a public charge; however, other utilized public health benefits may still count against citizenship eligibility.^39,47^ Lack of clarity around this issue may perpetuate health disparities among undocumented immigrants.

In addition, fears related to the Public Charge Rule create challenges for existing local programs. According to our participants, undocumented immigrants are increasingly less likely apply to Medi-Cal, fearing deleterious consequences from disclosure of personal information. When undocumented immigrants fail to obtain Medi-Cal disapproval, they become ineligible for nearly all LA County programs—even those not affected by the Public Charge Rule. For families experiencing poverty, especially those with chronic conditions, lack of program assistance can become a cost-prohibitive deterrent to health care engagement. The health system currently has limited ability to address such dramatic implications of the new Public Charge Rule, especially for such a large population. Additional study to quantify the rule's effect on health costs and outcomes will be critical to informing federal lawmakers who revisit the policy going forward.

At a health system level, participants interviewed identified a complex network of programs and partnerships, each with distinct eligibility criteria and enrollment procedures—all making for difficult navigation. Because undocumented immigrants are ineligible for federal programs, programs that fill “coverage gaps” for community populations will not only remain essential but will also continue to be subject to significant bureaucratic enrollment requirements. Local health systems, therefore, have a great opportunity to improve access for undocumented immigrants by enhancing navigation of these services.

Immigration policy changes that penalize those who need care may also erode patient trust in the health system. Trust is essential, particularly for safety-net health systems that are often the only option for care among undocumented immigrants.^48^ If patient trust is displaced by trepidation, suspicion, and fear, we anticipate a growing “chilling effect” of health service utilization.^2,45,48,49^ We found health care workers in LA County felt incapable of addressing misconceptions and false information disseminated throughout the community. Additional investment of health system resources to educate the public may reduce misconceptions and increase patient trust.

At an individual level, misinformation and misperceptions were exacerbated by communication and socioeconomic challenges—especially language proficiency and literacy. Although such challenges are not new, health care worker participants pointed out current approaches to address them still fall short, exacerbating impacts of immigration and coverage policies. Novel and innovative approaches to address these barriers could greatly improve access. For example, mobile/virtual technology within safety-net health care settings may be tailored to patients at varying levels of literacy and different backgrounds.^50–53^ In LA County, patient access to specialty services dramatically increased with the adoption of e-consult practices.^54^ Similarly, using virtual care to address nonurgent primary care complaints may circumvent patient fears and improve access to care. These types of interventions—which may greatly accelerate in the era of COVID-19—will enable health care systems working within federal policy constraints to better deliver care to diverse patient populations.

We note several limitations in our study. First, perspectives of health care workers are often anecdotal and do not represent or replace the voices of undocumented immigrants unable to access care, and perceptions may be biased. Second, Latinx immigrants comprise a majority of the population, and as such, their concerns and needs dominated participant feedback. Third, whereas the number of uninsured in LA County is well established, the number with incomes <138% of the federal poverty level is not established, and the number of uninsured who are eligible for My Health LA is likely less. Fourth, health care worker interviews represent two of four county hospitals and a sampling of community clinics, and perceptions/practices and patient populations elsewhere may differ. Fifth, qualitative information about appointment availability is limited in the absence of appointment data. Finally, although LA County represents only one metropolitan undocumented immigrant population, findings align with those reported in other communities and provide insights into how barriers to care in similar settings may potentially be mitigated.

## Conclusion

Ensuring access to care for undocumented immigrants in the United States is a significant challenge. In LA County, where novel programs effectively remove barriers such as the cost of coverage and appointment availability, providers observe limited access to care among undocumented immigrants due to fear and health system navigation complexities. Federal immigration policy changes—especially the Public Charge Rule—may lead to prohibitively high risks for engaging in health care programs, resulting in unnecessary delays in care, higher health spending, and poorer outcomes. Misinformation about the availability of county health care programs that do not apply to the Public Charge Rule definition may further deter patients from engaging in care. Empirical evaluation of this policy's impact represents an essential area for future study, especially as it may inform subsequent revision to the policy. Investing in patient navigation assistance and novel approaches to improve health care system capacity to address these challenges may be beneficial and represent important areas for future study.

## Abbreviations Used

ACA: Affordable Care Act
CCALAC: Community Clinic Association of Los Angeles County
COVID-19: coronavirus disease 2019
FQHC: federally qualified health center
ICE: Immigration and Customs Enforcement
LA County: Los Angeles County
LAC-DHS: Los Angeles County Department of Health Services
MHLA: My Health LA
